# Metagenome Sequence of a Microbial Community from the Gold Mine Tailings in the Kuzbass Area, Russia

**DOI:** 10.1128/genomeA.01355-17

**Published:** 2017-12-07

**Authors:** Andrey V. Mardanov, Alexey V. Beletsky, Denis A. Ivasenko, Olga V. Karnachuk, Nikolai V. Ravin

**Affiliations:** a Institute of Bioengineering, Research Center of Biotechnology of the Russian Academy of Sciences, Moscow, Russia; b Laboratory of Biochemistry and Molecular Biology, Tomsk State University, Tomsk, Russia

## Abstract

The metagenome of a microbial community of the sediments from a highly acidic iron-rich puddle at the tailings dump of the Komsomolskaya gold mine in the Kuzbass area, Siberia, Russia, was sequenced. Binning of contigs yielded a near-complete genome of the dominant bacterium, representing a novel deep lineage of *Deltaproteobacteria*.

## GENOME ANNOUNCEMENT

Acid mine drainage (AMD) is formed as a result of the oxidation of pyrite and other sulfide minerals in the presence of oxygen and water (1). Under conditions of high acidity (pH 1 to 4), metal-containing minerals became dissolved. Mine waste and tailing dump sites are often characterized by AMD with high metal and sulfate concentrations. They represent extreme ecosystems harboring specific microbial communities, usually characterized by low biodiversity (2). Microbiological and molecular studies have shown that these communities include iron- and sulfur-oxidizing chemolithoautotrophs, as well as various groups of heterotrophic bacteria and archaea (3–6).

The object of this study was to characterize the microbial community of the sediments in a small acidic puddle from a tailing dump of the Komsomolskaya gold mine in the Kemerovo region, Russia (55.634354N, 88.195423E). The water had an intense orange color due to the high iron concentration. It was acidic (pH 2.29) and oxidized (Eh, +529 mV) and had a temperature of 26°C. Chemical analysis of the water revealed high concentrations of metals and metalloids, including iron (6,088 mg/liter), arsenic (800 mg/liter), magnesium (385 mg/liter), aluminum (504 mg/liter), zinc (136 mg/liter), copper (25 mg/liter), manganese (16 mg/liter), nickel (7 mg/liter), lead (4 mg/liter), cobalt (4 mg/liter), and cadmium (3 mg/liter). High acidity resulted from the oxidation of residual sulfides, mostly pyrite, which is consistent with a high content of sulfates (sulfur content was 5,731 mg/liter).

Metagenomic DNA was isolated from the sediment sample using the PowerSoil DNA isolation kit (Mo Bio Laboratories, Carlsbad, CA, USA) and sequenced with a Roche Genome Sequencer FLX (GS FLX), using the Titanium XL+ protocol for a shotgun library. About 221 Mb of sequence, with an average read length of 505 nucleotides (nt), was obtained. The reads were *de novo* assembled into contigs using the Newbler Assembler version 2.9 (454 Life Sciences, Branford, CT). A total of 10,427 contigs longer than 500 bp with an *N*_50_ contig size of 1,898 bp were obtained. Contigs comprising 16S rRNA gene sequences were identified using CheckM (7). Taxonomic assignment of these sequences using a BLASTN search against the NCBI nonredundant (NR) database revealed the nine most abundant microbes to be *Acidithiobacillus*, *Leptospirillum*, *Ferrimicrobium*, *Acidibacter*, *Acidisphaera*, uncultured bacterial lineages assigned to *Actinobacteria* and *Deltaproteobacteria*, archaea of the genus *Ferroplasma*, and an uncultured archaeal lineage related to *Thermoplasmatales*.

Binning of contigs using the CONCOCT tool (8) allowed us to obtain a near-complete 2.1 Mbp-long composite genome of the dominant deltaproteobacterium, consisting of 321 contigs. Analysis of the 16S rRNA sequence of this genome revealed that this bacterium was phylogenetically distant from cultured species (less than 84% 16S rRNA sequence identity) and represented a novel deep lineage of *Deltaproteobacteria*. Highly similar 16S rRNA gene sequences have been reported for mining wastes (9). The obtained metagenomic data will be useful for further studies of microbial processes in AMD-associated environments and genome-based analysis of the novel lineage of *Deltaproteobacteria*.

### Accession number(s).

This BioProject has been deposited in NCBI under the accession number PRJNA414965. The sequences obtained in this project (sample KU3) have been deposited in the NCBI Sequence Read Archive under the accession number SRR6189722.
